# Sex differences in the effects of lifetime intellectual cognitive reserve on language trajectory and risk of non-amnestic mild cognitive impairment: a longitudinal study

**DOI:** 10.1007/s10433-026-00927-8

**Published:** 2026-07-29

**Authors:** Dandan Wang, Mingxi Dang, Shijie Long, Jun Wang, Xiaoyi Hu, Xin Li, Zhanjun Zhang

**Affiliations:** 1 Faculty of Education, Beijing Normal University, Beijing, 100875 China; 2 State Key Laboratory of Cognitive Neuroscience and Learning, Faculty of Psychology, Beijing Normal University, Beijing, 100875 China; 3 Beijing Aging Brain Rejuvenation Initiative (BABRI) Centre, Beijing Normal University, Beijing, 100875 China; 4 School of Systems Science, Beijing Normal University, Beijing, 100875 China; 5 Beijing Key Laboratory of Cognitive Intelligence for Elderly Brain Health, Faculty of Psychology, Beijing Normal University, Beijing, 100875 China; 6 Faculty of Education, School of Special Education, Beijing Normal University, Beijing, 100875 China; 7 Institute for Advanced Study, Beijing Normal University, Beijing, 100875 China; 8 Innovation Institute of Integrated Traditional Chinese and Western Medicine, Shandong First Medical University & Shandong Academy of Medical Sciences, Jinan, 250117 Shandong China

**Keywords:** Life course intellectual engagement factors, Cognitive trajectory, Language ability, Mild cognitive impairment risk, Sex difference

## Abstract

Our previous study had confirmed that lifetime intellectual cognitive reserve (LICR) is associated with better late-life cognition. However, whether LICR can delay cognitive decline remains unknown. We systematically examined the influence of LICR on cognitive trajectories, as well as mild cognitive impairment (MCI) risk, and whether such associations differ by sex. A total of 1308 cognitively intact individuals (mean age 65.54 years) were included. Information on early educational attainment, midlife occupational complexity and mental leisure activities after retirement collected at baseline was used to construct LICR, while cognitive domain scores were derived from several neuropsychological tests assessed at baseline and twice follow-up, using confirmatory factor analysis. Data were analyzed using linear mixed-effects models and Cox regression models. Compared to participants with lower LICR, those with higher LICR exhibited significantly slower language ability decline (*β* = 0.071, 95% CI 0.002–0.14, *p* = 0.043), reduced risk of MCI (HR = 0.44, 95% CI 0.23–0.85, *p* = 0.014) and non-amnestic MCI (naMCI) (HR = 0.09, 95% CI 0.03–0.34, *p* < 0.001), rather than aMCI. Examination of its components showed that higher educational attainment was related to slower language ability decline (*β* = 0.014, 95% CI 0.004–0.02, *p* = 0.004), and it was also associated with reduced risk of naMCI (HR = 0.84, 95% CI 0.74–0.94, *p* = 0.004). Analyses by sex revealed that women with higher LICR had slower language ability decline (*β* = 0.04, 95% CI 0.01–0.07, *p* = 0.003). Among older adults, especially women, higher LICR was associated with preserved language ability and a lower risk of naMCI.

**Supplementary Information:**

The online version contains supplementary material available at https://doi.org/10.1007/s10433-026-00927-8.

## Introduction

With the accelerating progress of population aging and increased risks of dementia, maintaining high cognitive function in older adults is important for the sustainable future of our aging societies. Current research on cognitive decline or dementia risk factors has focused on variables that reduce the likelihood of cognitive symptoms (Silva et al. 2019; Arora et al. 2024). The cognitive reserve (CR) is one such variable that can increase resilience against cognitive decline and even Alzheimer’s disease, helping to maintain cognitive function (Stern et al. 2023, 2020; Stern 2002; Nelson et al. 2021; Chapko et al. 2018). Since CR is a dynamic construct, one proxy indicator during a certain time period alone cannot fully explain the accumulation of protective factors over the life course. Therefore, it is more valuable to define the CR by using several indicators covering different periods of life, especially intellectual engagement factors, which have shown greater protective advantages (Petkus and Gomez 2021; Hertzog et al. 2010). Our previous study also confirmed that greater intellectual engagement over the life course, called the lifetime intellectual cognitive reserve (LICR), which includes educational attainment, occupational complexity and mental leisure activities, is associated with better cognitive function, especially executive processing ability (Wang et al. 2024). Generally speaking, lifetime intellectual cognitive reserve is similar to living an “intellectually stimulating life”. To date, evidence on whether an intellectually active lifestyle helps to delay cognitive decline, or even reduce the risk of mild cognitive impairment (MCI) in cognitively intact individuals, is still limited.

Most current studies have focused on exploring the relationships between individual CR and cognitive decline and have shown inconsistent results. High educational attainment can preserve memory, verbal fluency and general cognitive ability (Foverskov et al. 2018), and a high level of occupational cognitive requirements is associated with a slower rate of cognitive decline after retirement (Pool et al. 2016). However, meta-analysis results revealed that the effect of educational attainment on delaying memory impairment and the decline of other cognitive functions was weak (Lvdén et al. 2020), and even the observed positive effect did not reach statistical significance (Seblova et al. 2020). Intellectual engagement in later life did not influence the trajectory of age-related cognitive decline (Staff et al. 2018). The abovementioned contradictory results further emphasize that CR has dynamic and comprehensive characteristics. Researchers have reported that higher occupational complexity combined with higher levels of cognitive activity are associated with reduced memory decline (Andel et al. 2016). Among individuals with low educational attainment and a less cognitively complex occupation, more mid-/late-life cognitive activity is associated with greater longitudinal global cognitive retention (Vemuri et al. 2014), which suggests that a higher comprehensive life-course cognitive reserve may be more conducive to the maintenance of crystallized intelligence. However, whether the effect of the LICR on the rate of cognitive change is reflected in fluid or crystallized intelligence remains to be further explored. In addition, it is yet to be determined whether the impact of the LICR on cognitive decline is equal for everyone or whether there is specific protection for some older adults. Since women are more susceptible to cognitive impairment (Ewers 2020) and dementia (Laws et al. 2018), if the LICR could contribute to maintaining cognitive ability in women, this could provide valuable insights for early interventions in women with cognitive impairment and even dementia. Furthermore, effective female‑oriented strategies may further inform comparable approaches to mitigate such cognitive risks among men.

A more direct approach to exploring the relationship between the accumulation of intellectual cognitive reserve factors over the life course and cognitive impairment involves the use of longitudinal follow-up data. MCI is an intermediate condition between normal cognition and dementia and is associated with an increased risk of dementia (Petersen 2011; Xu et al. 2020). Previous studies have confirmed that high composite CR scores, including education, reading and vocabulary measures (Pettigrew et al. 2017), and high lifespan CR, encompassing education and early-life to late-life cognitive and social activities (Xu et al. 2020), are associated with a decreased risk of MCI. However, to date, no empirical studies have examined whether leading an intellectually stimulating life throughout one’s lifetime increases an individual’s cognitive stability or whether it may serve as a protective factor against MCI, and if so, whether it is beneficial for all subtypes of MCI.

The present study extends our prior work, aiming to examine the associations between LICR and changes in fluid and crystallized intelligence over time and exploring whether such associations differ by sex, as well as verifying the hypothesis that a high LICR is associated with a reduced risk of MCI, using data from a long-term community-based cohort study. Since actions to decrease the risk of cognitive impairment should begin early and continue throughout life, the present study may provide a reference for the development of early cognitive interventions.

## Methods

### Participants

**Fig. 1:**
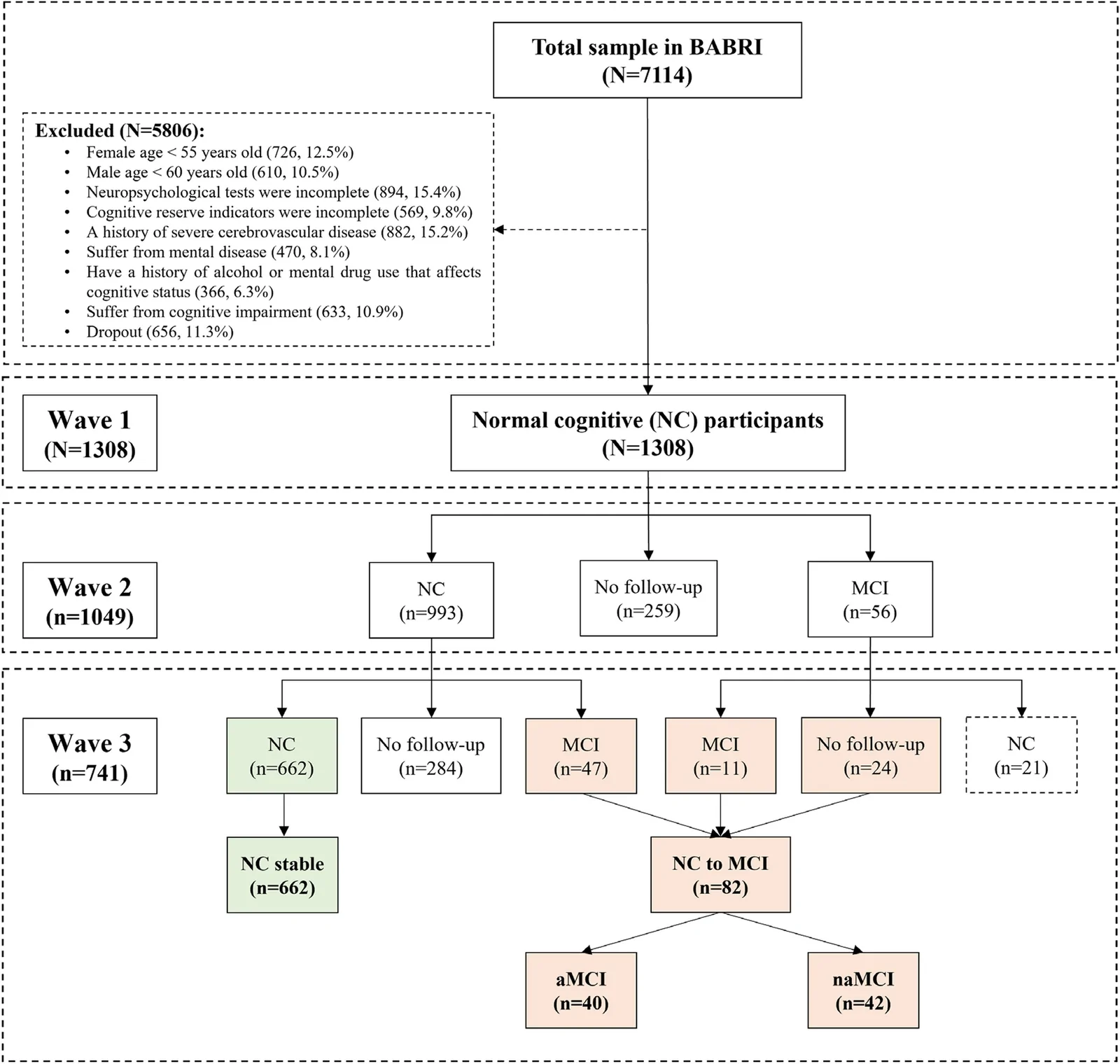
Flowchart of the study population. MCI = mild cognitive impairment; aMCI = amnestic mild cognitive impairment; naMCI = non-amnestic mild cognitive impairment

The Beijing Aging Brain Rejuvenation Initiative (BABRI) is an ongoing community-based prospective cohort study in China (Yang et al. 2021). A total of 1308 cognitively intact participants were enrolled at baseline. Across the two follow-up visits we examined, 82 participants developed MCI (24 at follow-up 1, mean follow-up, 2.37 ± 0.93 years after baseline; 58 at the second follow-up, mean follow-up, 3.35 ± 1.06 years after follow-up 1). The detailed information is shown in Fig. 1 and supplementary results in Supplementary file1.

All participants met the following criteria: no dementia; were native Chinese speakers; were retired (women at 55 years old, men at 60 years old); and had no history of neurologic, psychiatric, or systemic illnesses known to influence cerebral function. All participants provided written informed consent for our protocol, which was approved by the ethics committee of the State Key Laboratory of Cognitive Neuroscience and Learning, Beijing Normal University.

The diagnosis of MCI was based on Petersen’s criteria: having subjective cognitive complaints, having cognitive impairment in at least one domain (lower than 1.5 SD below the age- and education-adjusted mean on both tests in any domain) but normal daily living ability. Specifically, aMCI was diagnosed based on memory impairment, while naMCI was diagnosed based on intact memory and the impairment of at least one other cognitive domain (i.e., visual ability, language, and/or executive function). The cognitive performances of normal cognition participants were within 1.5 SDs (Petersen and Morris 2005; Yang et al. 2022; Du et al. 2023).

### Assessment of lifetime intellectual cognitive reserve

Data on early-life educational attainment, midlife occupational complexity, and mental leisure activities after retirement were acquired at baseline, to construct lifetime intellectual cognitive reserve (LICR). Detailed descriptions of these indicators can be found in a previous study (Wang et al. 2024).

Educational attainment was calculated on the basis of the number of years of regular school reported. The total occupational complexity score was comprised of three dimensions reflecting the interactions between people (0–8), data (0–6) and things (0–7) that participants were involved in during their work process (Andel et al. 2016; Boots et al. 2015). The score was then reversed; that is, the higher the total score, the greater the occupational complexity (0–21). The participants completed a 23-item questionnaire to collect information on mental leisure activities. The frequency of participation in each activity was rated from 1 (never) to 5 (every day), and the total score was based on a weighted score of these items.

### Neuropsychological tests

All the investigators have undergone unified professional training. At three time points, the participants all underwent a battery of neuropsychological tests (Chen et al. 2019): (1) the Auditory Verbal Learning Test (AVLT, including N1N3, N4, N5 and N1N5); (2) the Rey‒Osterrieth Complex Figure Test (ROCF, including copy and delayed recall); (3) the Digit Span Test (DST, including forward and backward); (4) the Trail Making Test (TMT, including A time, B time and B-A time); (5) the Symbol Digit Modalities Test (SDMT); (6) the Stroop Color and Word Test (SCWT, including B time, C time and C-B time); (7) the Clock Drawing Test (CDT); (8) the Category Verbal Fluency Test (CVFT, including animal, vegetable and fruit); and (9) the Chinese version of the Boston Naming Test (BNT) (Guo et al. 2006).

### Assessment of covariates

Empirical evidence suggests that addressing many risk factors, including hypertension, smoking, obesity, depression, physical inactivity, diabetes, excessive alcohol consumption, social isolation, high low-density lipoprotein cholesterol and others, could reduce the risk of developing dementia by up to 45% (Livingston et al. 2024). To examine the relationships between the LICR and cognition and the risk of MCI alone, these variables were used as covariates in subsequent statistical analyses.

Information on a broad range of these covariates was collected at baseline, including age, sex, emotional status (loneliness and depression), lifestyle factors (smoking [no/yes], drinking [no/yes], and physical activity), body mass index (BMI, kg/m^2^; continuous), chronic disease (hypertension [no/yes], diabetes [no/yes], hyperlipidemia [no/yes], coronary heart disease [no/yes], and cerebral small-vessel disease [no/yes]). The collection of these covariates is detailed in Supplementary file1.

### Statistical analysis

The variable characteristics of different groups were compared using *t* tests, *χ*^*2*^ tests or ANOVA, as appropriate. Exploratory factor analysis (EFA) and confirmatory factor analysis (CFA, Mplus8.3) were performed via the cross-validation method to derive cognitive domain scores according to the 9 neuropsychological tests. To place different tests on a comparable scale, all raw scores were standardized and reaction‑time‑related scores were reverse‑coded. Educational attainment was not adjusted for, given that it constituted a core component of LICR and was part of the exposure of interest, rather than a confounder requiring exclusion from cognitive outcome analyses. The model fit effect was assessed using chi-square (*χ*^2^) goodness-of-fit, the comparative fit index (CFI), the Tucker-Lewis index (TLI), the root mean square error of approximation (RMSEA), and the standardized root mean square residual (SRMR). Another CFA was performed to derive the predicted values of the LICR on the basis of the weights of the three indicators. In addition to using the continuous variable, we also utilized the distribution characteristics of LICR to divide the sample into three groups based on tertiles. That is, LICR was sorted in ascending order and split at the 33rd and 66th percentiles, finally yielding three groups: the highest, middle, and lowest LICR groups.

Linear mixed-effects models (SPSS 26.0) were used to estimate the β coefficients of the associations between the LICR and annual changes in several cognitive functions, with follow-up time (in years) as the time scale. Time was measured as the difference between the Wave 1 and Wave 2 interviews and the Wave 2 and Wave 3 interviews. The fixed effects included the LICR, time, and their interaction; to allow for individual differences at baseline and over time, we also included random effects for the intercept and slope for time in the model (Li et al. 2021), with the above confounders at baseline considered covariates. When the LICR was used as a continuous variable, each point change in cognitive function was associated with a unit change in the LICR. When the LICR was used as a categorical variable, each point change in cognitive function corresponded to the middle or the highest LICR in comparison to the lowest one. To further explore whether the relationship between the LICR and cognitive changes differs by sex, and whether the LICR plays a greater role in delaying cognitive decline than does any of its individual components, we conducted the aforementioned linear mixed-effects models separately for both men and women, and for each component of the LICR (continuous variable).

Furthermore, the multi-adjusted Cox regression models (SPSS 26.0) were used to estimate the hazard ratios (HRs) and 95% CIs for MCI (including aMCI and naMCI) incidence associated with the LICR and its components, with the above confounders at baseline considered covariates. The follow-up time was calculated as the time from study entry to MCI diagnosis or the last examination.

## Results

### Characteristics of the study population

At baseline, all 1308 participants were cognitively intact, 909 (69.5%) were women, and the mean (SD) age was 65.54 (6.07) years. Table 1 and eTable 1 present the characteristics of the study population. The characteristics of the participants who experienced MCI and were stable NCs at the second follow-up are presented in eTable 2 in Supplementary file1.

**Table 1:** Descriptive statistics at baseline

	Wave 1 *n* = 1308	Highest *n* = 432	Middle *n* = 432	Lowest *n* = 444	*F/χ*^*2*^	*p*	*η*^*2*^
Lifetime Intellectual Cognitive Reserve (LICR) ^a^	0.00 (− 0.91–0.91)	1.42 ± 0.58	− 0.04 ± 0.36	− 1.34 ± 0.53	1455.03	< 0.001	0.83
Early-Educational Attainment (EA) ^a^	11.58 ± 2.93	14.59 ± 1.88	11.26 ± 1.67	8.97 ± 1.87	418.18	< 0.001	0.59
Middle-Occupational Complexity (OC) ^a^	6.71 ± 3.89	10.11 ± 2.68	7.03 ± 2.64	3.08 ± 2.49	361.55	< 0.001	0.55
Later-Mental Leisure Activity (MLA) ^a^	62.09 ± 22.23	73.39 ± 22.65	61.66 ± 20.16	51.51 ± 18.09	55.18	< 0.001	0.16
Cognitive Function							
Working Memory (WM) ^a^	0.00 (− 0.68–0.77)	0.30 ± 1.07	0.04 ± 1.00	− 0.33 ± 1.01	16.10	< 0.001	0.05
DST-forwards ^a^	7.75 ± 1.27	8.01 ± 1.21	7.84 ± 1.20	7.41 ± 1.32	9.18	< 0.001	0.03
DST-backwards^a^	4.64 ± 1.30	4.99 ± 1.38	4.64 ± 1.27	4.31 ± 1.15	13.33	< 0.001	0.04
Episodic Memory (EM)^a^	0.00 (− 1.83–1.63)	0.38 ± 2.47	0.07 ± 2.39	− 0.43 ± 2.18	10.55	< 0.001	0.03
AVLT-N1N3^a^	18.53 ± 4.36	19.41 ± 4.49	18.50 ± 4.33	17.70 ± 4.10	11.36	< 0.001	0.04
AVLT-N4^a^	6.35 ± 2.10	6.59 ± 2.18	6.47 ± 2.11	6.00 ± 1.96	8.40	< 0.001	0.03
AVLT-N5^a^	5.86 ± 2.18	6.13 ± 2.26	5.91 ± 2.22	5.53 ± 2.02	6.58	0.001	0.02
Processing Speed (PS)^a^	0.00 (− 0.61–0.77)	0.25 ± 0.95	0.05 ± 1.04	− 0.30 ± 1.28	20.82	< 0.001	0.07
TMT A time (s)^a^	54.73 ± 15.85	51.48 ± 13.71	54.04 ± 16.26	58.56 ± 16.61	9.78	< 0.001	0.03
TMT B time (s)^a^	157.31 ± 53.15	146.07 ± 44.97	155.00 ± 48.78	170.58 ± 61.24	19.02	< 0.001	0.06
TMT B-A time (s)^a^	102.73 ± 48.50	94.85 ± 41.46	101.05 ± 44.57	112.08 ± 56.48	13.30	< 0.001	0.04
Visuospatial Ability (VA)^a^	0.00 (− 0.44–0.54)	0.19 ± 0.82	0.03 ± 0.80	− 0.21 ± 0.95	13.61	< 0.001	0.04
RO-delay^a^	13.69 ± 6.13	15.00 ± 6.33	13.79 ± 5.64	12.32 ± 6.11	12.73	< 0.001	0.04
RO-copy^a^	34.07 ± 2.97	34.46 ± 2.55	34.16 ± 2.78	33.58 ± 3.45	4.84	0.008	0.02
Language Ability (LA)^a^	0.00 (− 0.90–0.85)	0.36 ± 1.38	− 0.01 ± 1.30	− 0.29 ± 1.25	15.24	< 0.001	0.05
CVFT-Animal^a^	18.25 ± 3.91	19.42 ± 3.81	18.10 ± 3.94	17.41 ± 3.74	13.36	< 0.001	0.04
CVFT-Vegetable^a^	15.40 ± 3.45	16.02 ± 3.60	15.41 ± 3.39	14.87 ± 3.28	8.53	< 0.001	0.03
CVFT-Fruit^a^	13.08 ± 3.00	13.51 ± 3.06	13.11 ± 3.02	12.69 ± 2.88	5.62	0.004	0.02
General Cognitive Ability (GCA)^a^	0.00 (− 2.92–2.83)	1.34 ± 3.92	0.18 ± 3.88	− 1.47 ± 4.02	36.42	< 0.001	0.11

The above values are represented by the mean ± SD. ^a^ Adjusted for age and sex. LICR categories: lowest group, − 3.41 to − 0.63; middle group, − 0.63 to 0.56; highest group, 0.56 to 3.47

For cognitive function, the best-fitting model of the CFA generated 5 factors, namely, working memory, episodic memory, processing speed, visuospatial ability and language ability. Then these five cognition scores subsequently predicted the general cognitive ability score. The predicted values of the latent variables were determined on the basis of the weights of their respective indicators as shown in eFig. 1 in Supplementary file1, and the model fit indices were as follows: *χ*^2^ = 133.84, df = 60, *p* < 0.001; RMSEA = 0.034; CFI = 0.978, TLI = 0.971; SRMR = 0.032. In addition, the cross-validation results are also presented in Supplementary file1.

### Construction of the latent variable model of the LICR

The LICR was generated on the basis of the SEM with the 3 CR-enhancing factors (early-life educational attainment, midlife occupational complexity, and late-life mental leisure activities), as shown in eFig. 2. The correlation coefficients among the 3 factors varied from 0.22 to 0.47, and the CFA model revealed that the three observed variables in the saturated model were all significant at the level of *p* < 0.001. A unique value for the LICR was obtained on the basis of the weights of the three factors for each participant, and the score ranged from − 3.41 to 3.47.

Among the 1308 participants, 432 (33%), 432 (33%), and 444 (34%) were included in the highest, middle, and lowest LICR groups, respectively. Compared with the participants in the lowest LICR group, the participants in the highest LICR group were older, less likely to be female, had lower depression scores, had healthier lifestyles, had higher LICR and its component scores, had better cognitive functions. There were no significant differences in terms of loneliness, drinking, BMI or chronic disease history among the 3 groups (Table 1).

### Relationship between the LICR and cognitive decline

We focused mainly on the relationships between the LICR and the baseline and the rates of change in these cognitive functions, and the results are shown in Table 2.

**Table 2:** β coefficients for the associations of LICR with baseline and changes in cognitive function over the follow-up period

	WM Estimate (95% CI)	EM Estimate (95% CI)	PS Estimate (95% CI)	VA Estimate (95% CI)	LA Estimate (95% CI)	GCA Estimate (95% CI)
Baseline^a^						
Continuous LICR	0.22 (0.17, 0.27)^***^	0.35 (0.24, 0.46)^***^	0.28 (0.23, 0.33)^***^	0.13 (0.09, 0.17)^***^	0.13 (0.07, 0.18)^***^	1.10 (0.92, 1.28)^***^
Categories LICR						
Highest	0.59 (0.44, 0.74)^***^	0.82 (0.50, 1.15)^***^	0.67 (0.52, 0.83)^***^	0.37 (0.25, 0.50)^***^	0.37 (0.21, 0.52)^***^	2.81 (2.27, 3.35)^***^
Middle	0.31 (0.17, 0.45)^***^	0.45 (0.14, 0.76)^**^	0.34 (0.19, 0.48)^***^	0.19 (0.07, 0.31)^**^	0.10 (-0.05, 0.25)	1.39 (0.87, 1.90)^***^
Lowest	Reference	Reference	Reference	Reference	Reference	Reference
Continuous EA	0.10 (0.08, 0.12)^***^	0.13 (0.08, 0.17)^***^	0.11 (0.09, 0.13)^***^	0.06 (0.04, 0.07)^***^	0.05 (0.03, 0.07)^***^	0.44 (0.37, 0.52)^***^
Continuous OC	0.03 (0.01, 0.04)^***^	0.07 (0.04, 0.10)^***^	0.05 (0.04, 0.07)^***^	0.02 (0.01, 0.03)^**^	0.02 (0.003, 0.04)^*^	0.18 (0.13, 0.24)^***^
Continuous MLA	0.01 (0.007, 0.02)^***^	0.02 (0.01, 0.03)^***^	0.01 (0.01, 0.02)^***^	0.01 (0.004, 0.01)^***^	0.01 (0.006, 0.01)^***^	0.07 (0.05, 0.08)^***^
Longitudinal^b^						
Continuous LICR × Time	− 0.004 (− 0.02, 0.02)	− 0.008 (− 0.05, 0.03)	− 0.01 (− 0.03, 0.01)	0.01 (− 0.005, 0.03)	0.03 (0.007, 0.05)^*^	0.02 (− 0.04, 0.08)
Categories LICR × Time						
Highest × Time	− 0.03 (− 0.08, 0.03)	− 0.02 (− 0.15, 0.11)	− 0.006 (− 0.07, 0.06)	0.03 (− 0.02, 0.08)	0.071 (0.002, 0.14)^*^	0.05 (− 0.15, 0.24)
Middle × Time	− 0.01 (− 0.07, 0.04)	− 0.02 (− 0.15, 0.10)	0.03 (− 0.03, 0.10)	0.04 (− 0.02, 0.09)	0.066 (− 0.001, 0.13)^+^	0.10 (− 0.09, 0.29)
Lowest × Time	Reference	Reference	Reference	Reference	Reference	Reference
Continuous EA × Time	− 0.002 (− 0.01, 0.01)	− 0.0002 (− 0.02, 0.02)	0.002 (− 0.01, 0.01)	0.004 (− 0.004, 0.01)	0.014 (0.004, 0.02)^**^	0.02 (− 0.01, 0.04)
Continuous OC × Time	0.0003 (− 0.01, 0.01)	− 0.003 (− 0.02, 0.01)	− 0.008 (− 0.01, − 0.001)^*^	0.004 (− 0.001, 0.01)	0.006 (− 0.001, 0.01)	0.0004 (− 0.02, 0.02)
Continuous MLA × Time	− 0.001 (− 0.002, 0.001)	− 0.001 (− 0.01, 0.002)	0.0003 (− 0.002, 0.002)	− 0.0001 (− 0.001, 0.002)	− 0.0004 (− 0.002, 0.002)	− 0.002 (− 0.007, 0.003)

^a^ Adjusted for baseline age (and age × time), sex, hypertension (and hypertension × time), diabetes (and diabetes × time), hyperlipidaemia (and hyperlipidaemia × time), coronary heart disease, cerebral small-vessel disease, smoking (and smoking × time), drinking (and drinking × time), BMI (and BMI × time), depression (and depression × time), loneliness (and loneliness × time) and physical activity (and physical activity × time). ^b^ Adjusted for the variables in ^a^ and corresponding baseline cognitive functions. Abbreviations: LICR, lifetime intellectual cognitive reserve; EA = educational attainment; OC = occupational complexity; MLA = mental leisure activity; WM = working memory; EM = episodic memory; PS = processing speed; VA = visuospatial ability; LA = language ability; GCA = general cognitive ability. ^***^ *p* < 0.001, ^**^ *p* < 0.01, ^*^ *p* < 0.05, ^+^ *p* < 0.06

For continuous indicators, participants with higher LICR were associated with better baseline cognitive functions. During the follow-up, episodic memory, language ability and general cognitive ability scores showed varying degrees of decline (the first two models in eTable 3). Participants with higher LICR were associated with slower decline in language ability by 0.03 (95% CI 0.007–0.05, *p* = 0.011) standard score units in multi-adjusted mixed-effects models. To verify the above results, the same analyses were conducted for the categorical LICR. The results revealed that participants in the highest and middle LICR groups had better baseline cognitive function in all domains than did those in the lowest LICR group. During the follow-up, compared with the lowest LICR group, participants in the highest LICR group were associated with slower decline in language ability by 0.071 (95% CI 0.002–0.14, *p* = 0.043) standard score units; participants in the middle LICR group showed a trend toward slower decline in language ability by 0.066 (95% CI − 0.001–0.13, *p* = 0.054) standard score units (eFig. 3).

For the components of the LICR, during the follow-up, participants with higher educational attainment were associated with slower decline in language ability by 0.014 (95% CI 0.004–0.02, *p* = 0.004) standard score units; participants with higher occupational complexity were associated with faster decline in processing speed by  − 0.008 (95% CI − 0.01–− 0.001, *p* = 0.029) standard score units; there was no significant relationship between mental leisure activity and the rate of decline in any of the cognitive domains (Table 2).

### Sex differences in the relationship between the LICR and cognitive decline

As shown in Table 3, at baseline, although the positive impact of the LICR on processing speed in men (*β* = 0.32, 95% CI 0.23–0.40, *p* < 0.001) was greater than that in women (*β* = 0.25, 95% CI 0.19–0.31, *p* < 0.001), the positive impacts of the LICR on episodic memory (*β* = 0.40, 95% CI 0.26–0.53, *p* < 0.001) and visuospatial ability (*β* = 0.13, 95% CI 0.08–0.18, *p* < 0.001) in women were greater than those in men (episodic memory: *β* = 0.30, 95% CI 0.11–0.49, *p* = 0.002; visuospatial ability: *β* = 0.11, 95% CI 0.02–0.19, *p* = 0.011). During the follow-up, only women with higher LICR were associated with a slower decline in language ability by 0.04 (95% CI 0.01–0.07, *p* = 0.003) standard score units. For the categorical LICR, only women in the highest LICR group were still associated with a slower decline in language ability by 0.11 (95% CI 0.03–0.19, *p* = 0.009) standard score units.

**Table 3:** Sex differences in β coefficients for the associations of LICR with baseline and changes in cognitive function over the follow-up period

	WM Estimate (95% CI)	EM Estimate (95% CI)	PS Estimate (95% CI)	VA Estimate (95% CI)	LA Estimate (95% CI)	GCA Estimate (95% CI)
Men						
Baseline^a^						
Continuous LICR	0.24 (0.15, 0.34)^***^	0.30 (0.11, 0.49)^**^	0.32 (0.23, 0.40)^***^	0.11 (0.02, 0.19)^*^	0.13 (0.04, 0.23)^**^	1.09 (0.77, 1.42)^***^
Categories LICR						
Highest	0.55 (0.24, 0.85)^***^	0.69 (0.12, 1.26)^*^	0.74 (0.47, 1.00)^***^	0.22 (− 0.02, 0.47)	0.31 (0.03, 0.60)^*^	2.52 (1.53, 3.51)^***^
Middle	0.16 (− 0.14, 0.46)	0.36 (− 0.20, 0.92)	0.33 (0.07, 0.59)^*^	− 0.04 (− 0.28, 0.21)	− 0.10 (− 0.38, 0.18)	0.73 (− 0.25, 1.70)
Lowest	Reference	Reference	Reference	Reference	Reference	Reference
Longitudinal^b^						
Continuous LICR × Time	− 0.02 (− 0.05, 0.02)	− 0.004 (− 0.08, 0.08)	− 0.009 (− 0.05, 0.03)	0.03 (− 0.008, 0.06)	0.01 (− 0.04, 0.05)	0.009 (− 0.11, 0.13)
Categories LICR × Time						
Highest × Time	− 0.05 (− 0.16, 0.06)	− 0.01 (− 0.25, 0.23)	0.03 (− 0.10, 0.15)	0.07 (− 0.03, 0.17)	0.007 (− 0.13, 0.14)	0.04 (− 0.32, 0.40)
Middle × Time	− 0.01 (− 0.13, 0.10)	− 0.04 (− 0.29, 0.20)	0.03 (− 0.09, 0.15)	0.04 (− 0.06, 0.15)	0.09 (− 0.05, 0.23)	0.10 (− 0.27, 0.48)
Lowest × Time	Reference	Reference	Reference	Reference	Reference	Reference
Women						
Baseline^a^						
Continuous LICR	0.21 (0.16, 0.27)^***^	0.40 (0.26, 0.53)^***^	0.25 (0.19, 0.31)^***^	0.13 (0.08, 0.18)^***^	0.13 (0.07, 0.20)^***^	1.12 (0.90, 1.34)^***^
Categories LICR						
Highest	0.59 (0.41, 0.77)^***^	1.04 (0.64, 1.43)^***^	0.59 (0.42, 0.76)^***^	0.37 (0.22, 0.52)^***^	0.39 (0.20, 0.58)^***^	2.96 (2.31, 3.62)^***^
Middle	0.36 (0.19, 0.52)^***^	0.59 (0.22, 0.96)^**^	0.32 (0.16, 0.48)^***^	0.25 (0.11, 0.39)^***^	0.19 (0.02, 0.37)^*^	1.73 (1.12, 2.34)^***^
Lowest	Reference	Reference	Reference	Reference	Reference	Reference
Longitudinal^b^						
Continuous LICR × Time	0.001 (− 0.02, 0.02)	− 0.01 (− 0.06, 0.04)	− 0.01 (− 0.04, 0.02)	0.01 (− 0.01, 0.03)	0.04 (0.01, 0.07)^**^	0.03 (− 0.05, 0.11)
Categories LICR × Time						
Highest × Time	− 0.02 (− 0.09, 0.05)	− 0.03 (− 0.19, 0.12)	− 0.01 (− 0.09, 0.07)	0.02 (− 0.05, 0.08)	0.11 (0.03, 0.19)^**^	0.06 (− 0.17, 0.28)
Middle × Time	− 0.02 (− 0.09, 0.04)	− 0.02 (− 0.16, 0.13)	0.03 (− 0.04, 0.11)	0.03 (− 0.03, 0.09)	0.05 (− 0.02, 0.13)	0.08 (− 0.14, 0.29)
Lowest × Time	Reference	Reference	Reference	Reference	Reference	Reference

^a^ Adjusted for baseline age (and age × time), sex, hypertension (and hypertension × time), diabetes (and diabetes × time), hyperlipidaemia (and hyperlipidaemia × time), coronary heart disease, cerebral small-vessel disease, smoking (and smoking × time), drinking (and drinking × time), BMI (and BMI × time), depression (and depression × time), loneliness (and loneliness × time) and physical activity (and physical activity × time). ^b^ Adjusted for the variables in ^a^ and corresponding baseline cognitive functions. Abbreviations: LICR = lifetime intellectual cognitive reserve; WM = working memory; EM = episodic memory; PS = processing speed; VA = visuospatial ability; LA = language ability; GCA = general cognitive ability. ^***^ *p* < 0.001, ^**^ *p* < 0.01, ^*^ *p* < 0.05

For the components of the LICR, during the follow-up, only women with higher educational attainment (*β* = 0.019, 95% CI 0.008–0.03, *p* = 0.001) were associated with a slower decline in language ability (eTable 4).

### Association of LICR with the risk of different subtypes of MCI

According to the multivariate Cox regression models, participants with higher LICR were associated with reduced risk of MCI (HR = 0.82, 95% CI 0.67–0.99, *p* = 0.043) and naMCI (HR = 0.66, 95% CI 0.50–0.87, *p* = 0.003) (Table 4). The same analyses were conducted for the categorical LICR to verify the results. Compared with the lowest LICR, the HR (95% CI) of MCI was 0.44 (0.23–0.85, *p* = 0.014), and the HR (95% CI) of naMCI was 0.09 (0.03–0.34, *p* < 0.001) for participants in the highest LICR group (Table 4 and eFig. 4). There was no significant relationship between LICR and the risk of aMCI, as shown in Supplementary file1.

**Table 4:** Hazard ratios (HRs) and confidence intervals (95% CIs) for the associations between LICR and the risk of different subtypes of MCI

LICR	No. of participants	No. of MCI	HR (95% CI)
Risk of MCI			
Continuous^a^	1287^b^	82^c^	0.82 (0.67–0.99)^*^
Components			
Early-Educational Attainment^a^	1287	82	0.93 (0.84–1.02)
Middle-Occupational Complexity^a^	1287	82	0.93 (0.87–0.99)^*^
Later-Mental Leisure Activity^a^	1287	82	1.01 (0.99–1.03)
Categorical (tertiles)^a^			
Highest	427	15	0.44 (0.23–0.85)^*^
Middle	427	23	0.62 (0.35–1.09)
Lowest	433	44	Reference
Risk of naMCI			
Continuous^a^	1298^d^	42^e^	0.66 (0.50–0.87)^**^
Components			
Early-Educational Attainment^a^	1298	42	0.84 (0.74–0.94)^**^
Middle-Occupational Complexity^a^	1298	42	0.93 (0.84–1.02)
Later-Mental Leisure Activity^a^	1298	42	0.99 (0.97–1.02)
Categorical (tertiles)^a^			
Highest	429	5	0.09 (0.03–0.34)^***^
Middle	431	15	0.56 (0.27–1.14)
Lowest	438	22	Reference
Risk of aMCI			
Continuous^a^	1297^f^	40^ g^	0.91 (0.65–1.26)
Components			
Early-Educational Attainment^a^	1297	40	0.99 (0.87–1.14)
Middle-Occupational Complexity^a^	1297	40	0.92 (0.83–1.02)
Later-Mental Leisure Activity^a^	1297	40	1.02 (0.99–1.05)
Categorical (tertiles)^a^			
Highest	430	10	0.87 (0.33–2.25)
Middle	428	8	0.46 (0.18–1.18)
Lowest	439	22	Reference

^a^ Adjusted for sex, age, hypertension, diabetes, hyperlipidaemia, coronary heart disease, cerebral small-vessel disease, smoking, drinking, physical activity, depression, loneliness, BMI, and baseline cognitive functions, including working memory, episodic memory, processing speed, visuospatial ability, language ability and general cognitive ability. ^b^ The number of participants was different from that in Table 1 and Fig. 1 because we excluded 21 participants who converted from MCI to cognitively normal. ^c^ Number of participants included 24 in Wave 2 and 58 in Wave 3. ^d^ Number of participants after excluding the 10 participants who converted to cognitive normal from MCI. ^e^ Number of participants included 11 in wave 2 and 31 in Wave 3. ^f^ Number of participants after excluding the 11 participants who converted to cognitive normal from MCI. ^g^ Number of participants included 13 in wave 2 and 27 in Wave 3. ^***^ *p* < 0.001, ^**^ *p* < 0.01, ^*^ *p* < 0.05

Since the number of individuals with MCI was less than half of the total population, we did not perform Kaplan–Meier survival analysis, nor did we analyze sex differences in the relationship between the LICR and the risk of MCI. For the components of the LICR, participants with higher educational attainment (HR = 0.84, 95% CI 0.74–0.94, *p* = 0.004) were associated with reduced risk of naMCI and higher occupational complexity (HR = 0.93, 95% CI 0.87–0.99, *p* = 0.046) were associated with reduced risk of MCI (Table 4). There was no significant relationship between other components of the LICR and the risk of MCI, aMCI and naMCI, as shown in Supplementary file1.

## Discussion

In this community-based longitudinal study, we aimed to examine the associations between LICR and cognitive trajectories and the risk of MCI in older adults in China. The main findings include the following: (1) after controlling for potential confounding factors, older adults with the highest LICR had preserved language ability, whereas among the components of the LICR, only educational attainment was associated with preserved language ability, and its protective effect was less than that of the LICR; (2) older women with the highest LICR also had slower language ability decline; and (3) older adults with the highest LICR were associated with a 56% reduction in MCI risk, a 91% reduction in naMCI risk, and no significant association with aMCI risk. The reduction effect for its components was less than that of the LICR. These results clarify that the lifetime accumulation of ICR factors could help maintain crystallized intelligence in older adults, especially among the female population. Notably, the protective effect of LICR against MCI was driven by its association with naMCI, whereas LICR did not appear to exert a protective effect against aMCI.

To date, the majority of studies have consistently reported that older adults with higher educational attainment and occupational complexity (Cations et al. 2018), and intellectual cognitive reserve factor accumulation over the life course (Wang et al. 2024) may have better fluid and crystallized intelligence in later life. Some longitudinal studies that combined ICR-related factors revealed that early-life ICR factors combined with late-life ICR factors (Vemuri et al. 2014) and midlife ICR factors combined with late-life ICR factors (Andel et al. 2016) had cumulative protective effects on global cognitive function decline and memory decline. However, no empirical studies have examined whether leading an intellectually stimulating life throughout one’s lifetime means increasing cognitive stability. The present study constructed the LICR, which is based on early educational attainment, midlife occupational complexity, and mental leisure activities after retirement, covering CR factors throughout the life course. We found that, compared with its individual components, LICR has a cumulative advantage for cognitive decline. Specifically, participants with higher LICR showed milder changes in working memory, episodic memory and processing speed; displayed a trending slower decline in visuospatial ability and general cognitive ability; and only language ability demonstrated a modest yet statistically significant association with higher LICR. These findings are consistent with the viewpoint that the positive impact of cognitive reserve on cognitive function might be the result of the accumulation of knowledge and language skills (Kramer et al. 2004), which indicates that the positive impact of intellectual cognitive reserve factor accumulation over the life course on the maintenance of crystallized intelligence is more significant. On the other hand, researchers have confirmed that during cognitive aging, cognitive function may gradually transition from reliance on fluid intelligence to greater dependence on crystallized intelligence, thereby resulting in the accumulation of crystallized intelligence (Spreng and Turner 2019), especially under the positive influence of lifetime intellectual protective factors (Reuter-Lorenz and Park 2024).

In addition, we also explored the relationships between the components of the LICR and cognitive trajectories. The results first showed that higher educational attainment tended to correlate with maintained language ability, which is consistent with a previous study (Lvdén et al. 2020). We believe that it is precisely because of the crucial leading role of the level of education received in early life, mid- and late-life cognitive engagement offers additional, non-redundant protection against language aging—a key message for public health policy targeting older adults with limited early educational opportunities. However, high occupational complexity was related to a faster rate of processing speed decline, which might be due to the following two possible reasons. From the perspective of cognitive psychological mechanisms, higher complexity occupations may also carry high risks. For instance, occupations such as doctors often experience excessive stress and face various risk exposures, which may counteract or even outweigh the protective effects of the occupational complexity itself, thereby impairing the more basic processing speed and subsequently damaging other advanced cognitive functions. From a neurobiological perspective, processing speed is strongly linked to age-sensitive frontoparietal and dorsal attention networks (Eckert 2011). Individuals in cognitively demanding occupations may rely more on these systems across adulthood (Small et al. 2009), potentially leading to compensatory depletion with aging, thereby making age-related processing speed vulnerabilities more evident. It should be noted that the above explanations require further empirical investigation for verification.

Furthermore, evidence on whether the impact of LICR on cognitive decline is consistent for all or provides specific protection for some older adults is limited. Sex-stratified analyses are critical in finding the most efficacious interventions for each sex. Previous researchers have found that years of education were inversely associated with age at onset only in women (Giacomucci et al. 2022). Although researchers believe that education might act as a minor contributor to cognitive reserve in women, this might also imply the significance of the accumulation of ICR factors over the life course. Since women are more susceptible to cognitive impairment (Ewers 2020) and dementia (Laws et al. 2018; Subramaniapillai et al. 2021), clarifying whether intellectual cognitive reserve factors benefit women’s cognitive maintenance is conducive to improving their late-life quality and achieving health equity. Although researchers have reported that a greater level of comprehensive cognitive reserve is associated with greater preservation of crystallized intelligence in terms of general cognitive ability among women, they did not specify whether this protective effect was specifically for women (Vemuri et al. 2014). Our study found that women with higher LICR exhibited modestly slower language ability decline, which might be related to the existing sex differences in cognitive development in older age. For instance, men generally perform better in visuospatial processing tasks (Castro-Alonso et al. 2019), while women generally show better cognitive performance in language (McCarrey et al. 2016). The observed association may offer preliminary implications for understanding cognitive aging in older females, and may provide tentative clues for future research on targeted strategies to slow language ability decline.

Studies with longitudinal design found that clinically defined MCI was not a stable population, and some studies have found that up to 24–50% of MCI individuals would revert to normal cognition status (Canevelli et al. 2016; Malek-Ahmadi 2016). Therefore, we believe that the transformation of 21 older adults from MCI to normal cognition during the second follow-up might be a relatively normal cognitive change. Based on this, we explored the relationships between the components of the LICR and the risk of MCI and observed that high early-life educational attainment and midlife occupational complexity were individually associated with a reduced risk of MCI, which is in agreement with previous studies (Cations et al. 2018; Gao et al. 2017; Edwin et al. 2024). Furthermore, the reduction effect of the LICR was greater than that of the individual components. Our study captured the LICR on the basis of the life course, and the measure was weighted according to its contribution to the score. Thus, it is not surprising that the association of the accumulation of intellectual cognitive reserve factors over the life course with both MCI and naMCI was stronger than the association of the individual factors with MCI. Moreover, LICR was more beneficial in reducing the risk of naMCI in older adults, which might be related to the aforementioned finding that LICR was more conducive to preserving language ability. That is, LICR may be more beneficial in reducing the risk of non-memory-related MCI. Nevertheless, this requires further analyses for verification. From the perspective of neural mechanisms, some researchers have reported that greater composite cognitive reserves are associated with greater structural integrity in the frontal and parietal lobes, including the left middle frontal gyrus and inferior parietal lobule (Bartrés-Faz et al. 2009; Conti et al. 2021), which are also related to language access and language retrieval (Cheng et al. 2023; Price 2012). However, this protective mechanism also requires further empirical exploration in combination with neuroimaging data. In brief, these results argue that CR is a dynamic construct; one proxy indicator during a certain time period alone cannot fully explain the accumulation of ICR factors over the life course, and actions to decrease cognitive impairment risk should begin early and continue throughout life.

The early intervention proposed based on the concept of LICR has two meanings. Firstly, the concept of LICR suggests intellectual engagement over the life course. In early life, individuals, especially women, should actively strive to achieve a higher level of education. When they started their careers, they could seek out and engage in as many complex occupations as possible within the limits of their attained education. Even after retirement or in later life, they should still follow the principle of “use it or lose it”, continuously engaging in intellectual activities within their capacity to maintain better cognitive abilities. Since early educational attainment plays a more significant role in delaying the decline of language ability, especially among women, and in reducing the risk of naMCI. We believe that to gain greater benefits from lifetime intellectual engagement, it is crucial to pursue a higher level of education in early life. Secondly, given that the intellectual engagement in early and midlife for older adults remains immutable, continuously enriching late-life intellectual stimulation becomes especially important for further improving LICR, which in turn helps maintain cognitive function. Such intellectually stimulating activities can include but are not limited to cognitive training. Our research team has previously evaluated the effectiveness of computerized cognitive training (CCT) in improving the executive function of older adults and its underlying neural mechanisms (increased axial diffusivity in the left posterior thalamic radiation) (Dai et al. 2024). Therefore, we believe that engaging in various forms of intellectual activities in later life can serve as an effective way to enrich LICR, and it also can be an early intervention strategy for maintaining cognitive function. Especially, older adults with low LICR who have comorbid depression and a sedentary lifestyle may represent a subgroup in need of intensified, multimodal intervention in later life (cognitive training + physical activity + depression management). Moreover, the protective mechanism for cognitive maintenance should include but not be limited to positive neuroplasticity. We will conduct further intervention studies to verify this.

A notable strength of our study is the community-based prospective cohort with a relatively large sample size and two follow-up examinations. The use of latent variables could capture the accumulation of intellectual engagement factors over the life course and representative cognitive domains with common characteristics. Furthermore, when examining how intellectual engagement protects against cognitive decline, we tested for sex-specific population effects and parsed its impact on the risk of different subtypes of MCI. Nevertheless, the current study also has several limitations. First, our participants were volunteers rather than being randomly selected from the community, and they were all living in urban areas; thus, caution is needed when generalizing our findings to older adults living in rural areas and other cultures. Moreover, more replication studies in different areas and cultures were expected to yield consistent results, though our research findings are consistent with those in the USA or other cultures (Andel et al. 2016; Vemuri et al. 2014; Pettigrew et al. 2017). Second, since CR is a theoretical and hypothetical construct, well-defined and accepted measures are not yet available, and the statistical results obtained by other methods cannot be directly compared with our results. Third, the number of participants who progressed to MCI during the follow-up was too small to examine the sex differences between LICR and the risk of MCI (and different subtypes of MCI, including aMCI and naMCI). Finally, we did not include brain neuroimaging characteristics and were unable to investigate the neural mechanism by which LICR delays the decline in language ability and reduces the risk of MCI in older adults. As for the future prospects and directions, as it is not clear whether the role of genetic risk in the pathological development or susceptibility of Alzheimer’s disease differs between women and men, it is important when looking at gender effects to assess the effects on the potential importance of the APOE risk gene and other various aspects.

## Conclusions

The present study provides evidence that older adults with higher lifetime intellectual cognitive reserve encompassing early-life educational attainment, mid-life occupational complexity and late-life mental leisure activity are associated with preserved language ability and reduced risk of naMCI, and the protective effect of the LICR is greater than that of any of its components. Furthermore, the association between higher LICR and preserved language ability was more evident among older women. These findings underscore that maintaining intellectually stimulating activities across the lifespan was linked to preserved language ability and may reduce the risk of mild cognitive impairment. In particular, lifetime intellectual engagement among women may be associated with long-term cognitive maintenance and could be considered a potential lifestyle-related protective factor. Further large population-based longitudinal studies are needed to verify the consistent effect of LICR on slowing language ability decline, particularly in women, as well as to explore sex differences in its protective association with different MCI subtypes. Additionally, multimodal neuroimaging data should be utilized to identify the specific positive neural plasticity induced by cumulative LICR, which may underlie the maintenance of cognitive function in older adults.

## Supplementary Information

Below is the link to the electronic supplementary material.

Supplementary file1 (DOCX 397 KB)

## Data Availability

The datasets generated and/or analyzed during the current study are not publicly available due to not acquiring consent from participants to share datasets but are available from the corresponding author on reasonable request.
