# Supplementary material for: Sex differences in the effects of lifetime intellectual cognitive reserve on language trajectory and risk of non-amnestic mild cognitive impairment: a longitudinal study
Source: Eur J Ageing. 2026 Jul 29;23(1):43. doi: 10.1007/s10433-026-00927-8 (PMC13710482; doi:10.1007/s10433-026-00927-8)
Supplement: Supplementary file 1 [file 10433_2026_927_MOESM1_ESM.docx]

**Supplementary materials**

This supplementary material (including 4 tables and 4 figures) was supporting the methods and results for the manuscript “Sex Differences in the effects of lifetime intellectual cognitive reserve on language trajectory and risk of non-amnestic mild cognitive impairment: a longitudinal study” by Wang et al.

# Supplementary methods

## Assessment of potential confounders

Information on a broad range of potential confounders was collected at baseline. The scores of loneliness and depression were collected by the revised UCLA Loneliness Scale (UCLA) [1, 2] and the Geriatric Depression Scale [3, 4]. **Loneliness** was assessed using the revised UCLA Loneliness Scale (UCLA), a widely used and reliable self-report measure. The scale consists 20 items probing satisfaction with social relationships in the past two weeks. For example, “How often do you feel that there is no one you can turn to?” Each item is rated on a 4-point Likert-type scale, where 1 = Never, 2 = Rarely, 3 = Sometimes, and 4 = Often. Total UCLA scores are calculated by summing items and reverse coding where necessary so that higher scores correspond to higher loneliness, and the total scores can range from 20 to 80. **Depression** was assessed using the 30-item Geriatric Depression Scale (GDS). Each item was a yes/no question and a score of 0 or 1 could be assigned to each answer. The GDS score ranged from 0 to 30, with higher scores indicative of more severe depressive symptoms. **Physical activity**, including walking for exercise, gardening or yard work, calisthenics or general exercise, bicycle riding (including stationary bikes) and swimming or water exercises, was assessed by questionnaires. Frequencies of participation in each activity were rated from 1 (never) to 5 (every day), and the total score was based on a weighted score of these items [1]. Other lifestyle factors, including smoking, drinking, and body mass index, were ascertained by self-reported information. **Drinking and smoking status** were grouped into yes or no, based on the self-reported information. Weight and height were measured at study entry by trained staff. **Body mass index (BMI)** was calculated as weight in kilograms divided by height in meters squared. Whether the participants suffered from chronic diseases was also ascertained by self-reported information on medical conditions. **Hypertension** was ascertained based on systolic blood pressure ≥140 mm Hg or diastolic blood pressure ≥90 mm Hg measured by mercury sphygmomanometer, self-reported history of hypertension, or the use of antihypertension drugs. **Diabetes** was defined by any of the following situations: hemoglobin A1c ≥6.5%, fasting plasma glucose ≥126 mg/dL, random blood glucose ≥200 mg/dL, self-reported history of diabetes, and the use of diabetes medication. **Hyperlipidaemia** was defined by plasma total cholesterol ≥5.18 mmol/L, triacylglycerol ≥1.70 mmol/L, low-density lipoprotein ≥3.37 mmol/L, self-reported history of hypertension, or the use of lipid-lowering drugs. **Coronary heart disease and cerebral small-vessel disease** were ascertained by self-reported information on medical conditions.

# Supplementary results

**Participants**

During the follow-up, 82 participants (24 at follow-up 1 and 58 at the second follow-up) developed MCI. Specifically, during follow-up 1, 993 participants maintained normal cognition (NC), 56 participants developed MCI (32 aMCI, 24 naMCI), and 259 participants dropped out. During follow-up 2, for the NC participants, 662 participants were still NC; 47 participants developed MCI (19 aMCI; 28 naMCI); and 284 participants dropped out; for the MCI participants, 21 participants converted to normal cognition, 11 participants were MCI stable (8 aMCI, 3 naMCI), and 24 MCI (13 aMCI, 11 naMCI) participants dropped out. The detailed information is shown in Fig. 1.

**Construction of latent variable model of cognitive function**

The cross-validation factor analysis was performed in baseline 1308 participants. The exploratory factor analysis (EFA) was randomly in 643 participants, and the model generated 5 factors. In the factor 1, AVLT-N1N3, N4 and N5 were named as episodic memory factors; in the factor 2, DST-forward and DST-backward were named as working memory; in the factor 3, CVFT-Animal, CVFT-Vegetable and CVFT-Fruit were named as language ability; in the factor 4, RO-copy and RO-delay were named as visuospatial ability; in the factor 5, TMT A time, TMT B time and TMT B-A time were named as processing speed. The five factors ultimately constituted the latent variable of general cognitive ability. The model fit indexes were as follows: 𝜒²=42.62, *df*=32, *p*=0.09; RMSEA=0.026; CFI=0.993; TLI=0.982, SRMR=0.024. A subsequent confirmatory factor analysis (CFA) included the remaining 665 participants. All variables in the above five factors generated were successfully incorporated into the model, and the model fit indexes were as follows: 𝜒²=84.22, *df*=42, *p*<0.001; RMSEA=0.044; CFI=0.970; TLI=0.945, SRMR=0.036.

Then, CFA was performed on the total participants, and the best-fitting model generated 5 factor scores and general cognitive ability score. All variables in the above factors generated were successfully incorporated into the model showed in the eFig. 1, with the fit indexes were as follows: χ^2^=133.84, df=60, *p*<0.001; RMSEA=0.034; CFI=0.978, TLI=0.971; SRMR=0.032.

**Characteristics of the NC stable and MCI participants in the second follow up**

During the second follow-up of the 1308 participants, total 82 participants stable developed to MCI, 50 (61.0%) were women and the mean (SD) age was 73.00 (6.21) years. F/t-tests on whether the older individuals developed to MCI revealed that those who stable progressed to MCI were older, had lower loneliness score, led less healthier lifestyles, suffered from more chronic diseases, had lower LICR and its components scores, and exhibited poorer cognitive performance. eTable 2 presented the demographic and cognitive characteristics of the NC stable and MCI participants.

**Association of LICR and its components scores with the risk of** **different subtypes of MCI**

According to the multivariate Cox regression models, a higher LICR was associated with a greater reduction in the risk of MCI (HR = 0.82, 95% CI 0.67 to 0.99, *p*=0.043) and naMCI (HR = 0.66, 95% CI 0.50 to 0.87, *p*=0.003) (Table 4). There was no significant relationship between LICR and the risk of aMCI (HR = 0.91, 95% CI 0.65 to 1.26, *p*=0.55). The same analyses were conducted for the categorical LICR to verify the results. Compared with the lowest LICR group, the HR (95% CI) of MCI was 0.44 (0.23 to 0.85, *p*=0.014), the HR (95% CI) of naMCI was 0.09 (0.03 to 0.34, *p*<0.001), and the HR (95% CI) of aMCI was 0.87 (0.33 to 2.25, *p*=0.77) for participants in the highest LICR group; the HR (95% CI) of MCI was 0.62 (0.35 to 1.09, *p*=0.095), the HR (95% CI) of naMCI was 0.56 (0.27 to 1.14, *p*=0.11), and the HR (95% CI) of aMCI was 0.46 (0.18 to 1.18, *p*=0.11) for participants in the middle LICR group.

To compare the advantage of the LICR and its components scores, we further explored the relationship between the three component indicators and the risk of MCI. In multi-adjusted Cox models (all component indicators were as continuous variable), higher occupational complexity (HR = 0.93, 95% CI 0.87 to 0.99, *p*=0.046) was associated with a reduction in the risk of MCI. There were no significant relationships between educational attainment (HR = 0.93, 95% CI 0.84 to 1.02, *p*=0.11) or mental leisure activity (HR = 1.01, 95% CI 0.99 to 1.03, *p*=0.48) and the risk of MCI.

For the risk of nonamnestic MCI (naMCI), in multi-adjusted Cox models, higher educational attainment (HR = 0.84, 95% CI 0.74 to 0.94, *p*=0.004) was associated with a reduction in naMCI risk. There were no significant relationships between occupational complexity (HR = 0.93, 95% CI 0.84 to 1.02, *p*=0.13) or mental leisure activity (HR = 0.99, 95% CI 0.97 to 1.02, *p*=0.64) and the risk of naMCI.

For the risk of amnestic MCI (aMCI), in multi-adjusted Cox models, there were no significant relationships between educational attainment (HR = 0.99, 95% CI 0.87 to 1.14, *p*=0.96), occupational complexity (HR = 0.92, 95% CI 0.83 to 1.02, *p*=0.11) or mental leisure activity (HR = 1.02, 95% CI 0.99 to 1.05, *p*=0.12) and the risk of aMCI.

# Supplementary figures

**eFig. 1 Standardized estimates from the CFA with 13 observable indicators of latent variables.**

*Note*. The values indicate the loading of the 13 factors to cognitive functions. e1, e2, ……and e13 indicate the measurement error for each cognitive function factor. The predicted values of the latent variables were derived via the maximum likelihood method and determined on the basis of the weights of their respective indicators.

**eFig. 2 Standardized estimates from the CFA with three observable indicators of a latent construct.**

*Note*. The values indicate the loadings of the three factors on the LICR. e1, e2, and e3 indicate the measurement error for each cognitive reserve factor. The composite indicator was determined on the basis of the weights of these factors.

**eFig. 3 The associations between LICR and the language trajectories by LICR group.**

*Note*. Shaded: 95% CI; cluster-robust by subject.

**eFig. 4 The cumulative risk of MCI and naMCI in different LICR group.**

# Supplementary tables

**eTable 1 Descriptive statistics of demographic and other characteristics at baseline.**

|  | **Wave 1**  **n=1308** | **Highest**  **n=432** | **Middle**  **n=432** | **Lowest**  **n=444** | ***F/χ²*** | ***p*** | ***η^2^*** |
| --- | --- | --- | --- | --- | --- | --- | --- |
| **Demographic** |  |  |  |  |  |  |  |
| Age | 65.54 ± 6.07 | 66.81 ± 6.40 | 64.74 ± 5.98 | 65.09 ± 5.60 | 8.88 | <0.001 | 0.03 |
| Sex (female, %) | 909 (69.5%) | 273 (63.2%) | 315 (72.9%) | 321 (72.3%) | 12.12 | 0.002 | / |
| **Emotional Status** |  |  |  |  |  |  |  |
| Depression (GDS) **^a^** | 7.37 ± 5.95 | 6.01 ± 4.84 | 7.97 ± 6.29 | 8.50 ± 6.20 | 8.23 | <0.001 | 0.03 |
| Loneliness (UCLA) **^a^** | 34.29 ± 9.12 | 33.33 ± 8.70 | 34.91 ± 9.03 | 34.66 ± 9.57 | 1.03 | 0.36 | 0.003 |
| **Lifestyle Factors** |  |  |  |  |  |  |  |
| Smoking (yes) | 216 (16.5%) | 67 (15.7%) | 58 (13.4%) | 91 (20.5%) | 8.18 | 0.017 | / |
| Drinking (yes) | 224 (17.1%) | 77 (17.8%) | 76 (17.6%) | 71 (16.0%) | 0.74 | 0.69 | / |
| Physical Activity **^a^** | 49.42 ± 16.34 | 54.03 ± 17.47 | 49.00 ± 15.91 | 45.34 ± 14.39 | 13.04 | <0.001 | 0.04 |
| **Body Mass Index (BMI) ^a^** | 25.03 ± 3.95 | 24.67 ± 3.68 | 25.04 ± 3.85 | 25.37 ± 4.27 | 2.95 | 0.053 | 0.01 |
| **Chronic Disease History** |  |  |  |  |  |  |  |
| Hypertension (yes) | 673 (51.4%) | 211 (48.8%) | 228 (52.8%) | 234 (52.7%) | 1.76 | 0.42 | / |
| Diabetes (yes) | 303 (23.2%) | 91 (21.1%) | 113 (23.6%) | 99 (22.3%) | 3.43 | 0.18 | / |
| Hyperlipidaemia (yes) | 603 (46.1%) | 185 (42.8%) | 197 (45.6%) | 221 (49.8%) | 4.32 | 0.12 | / |
| Coronary Heart Disease (yes) | 211 (16.1%) | 58 (13.4%) | 74 (17.1%) | 79 (17.8%) | 3.60 | 0.17 | / |
| Cerebral Small-Vessel Disease (yes) | 215 (16.4%) | 61 (14.1%) | 76 (17.6%) | 78 (17.6%) | 2.52 | 0.28 | / |

***Note.*** The above values are represented by the mean ± SD (IQR) or number (proportion %). ^a^ Adjusted for age and sex. Abbreviations: GDS = Geriatric Depression Scale; LS = Loneliness Scale. The missing data were as follows: smoking=25; drinking=148; CHD=1; GDS=179; UCLA=245; BMI=7; LA=172. For continuous variables, the missing values were replaced using the method of mean substitution. For categorical variables, the missing values were used as independent categories.

**eTable 2 Descriptive statistics of the NC stable and NC-MCI participants at baseline.**

|  | **NC stable**  **n=1205** | **NC-MCI**  **n=82** | ***t/F*** | ***p*** | ***η^2^*** |
| --- | --- | --- | --- | --- | --- |
| **Demographic** |  |  |  |  |  |
| Age | 68.94 ± 6.34 | 73.00 ± 6.21 | -5.73 | <0.001 | / |
| Sex (female, %) | 851 (70.6%) | 50 (61.0%) | 3.40 | 0.07 | / |
| **Emotional Status** |  |  |  |  |  |
| Depression (GDS) **^a^** | 8.92 ± 5.88 | 10.23 ± 5.56 | 3.12 | 0.08 | 0.003 |
| Loneliness (UCLA) **^a^** | 33.18 ± 9.04 | 26.00 ± 3.49 | 16.09 | <0.001 | 0.01 |
| **Lifestyle Factors** |  |  |  |  |  |
| Smoking (yes) | 185 (15.4%) | 23 (28.0%) | 9.34 | 0.002 | / |
| Drinking (yes) | 202 (16.8%) | 15 (18.3%) | 0.22 | 0.64 | / |
| Physical Activity **^a^** | 50.18 ± 16.39 | 45.09 ± 13.36 | 5.28 | 0.022 | 0.004 |
| **Body Mass Index (BMI) ^a^** | 24.71 ± 3.87 | 24.61 ± 3.89 | 0.08 | 0.93 | <0.001 |
| **Chronic Disease** |  |  |  |  |  |
| Hypertension (yes) | 612 (50.8%) | 51 (62.2%) | 4.00 | 0.046 | / |
| Diabetes (yes) | 277 (23.0%) | 23 (28.0%) | 1.10 | 0.29 | / |
| Hyperlipidaemia (yes) | 561 (46.6%) | 33 (40.2%) | 1.23 | 0.27 | / |
| Coronary Heart Disease (yes) | 193 (16.0%) | 16 (19.5%) | 0.68 | 0.41 | / |
| Cerebral Small-Vessel Disease (yes) | 192 (15.9%) | 20 (24.4%) | 3.99 | 0.046 | / |
| **Lifetime Intellectual Cognitive Reserve (LICR) ^a^** | 0.06 ± 1.24 | -0.52 ± 1.24 | 25.34 | <0.001 | 0.02 |
| Early-Educational Attainment (EA) **^a^** | 11.68 ± 2.90 | 10.36 ± 3.20 | 21.80 | <0.001 | 0.02 |
| Middle-Occupational Complexity (OC) **^a^** | 6.83 ± 3.89 | 5.85 ± 3.68 | 11.56 | 0.001 | 0.01 |
| Later-Mental Leisure Activity (MLA) **^a^** | 63.12 ± 22.24 | 55.40 ± 19.43 | 7.67 | 0.006 | 0.01 |
| **Cognitive Function** |  |  |  |  |  |
| **Working Memory (WM) ^a^** | 0.08 ± 1.02 | -0.33 ± 1.09 | 9.30 | 0.002 | 0.01 |
| DST-forwards **^a^** | 7.91 ± 1.29 | 7.67 ± 1.43 | 1.59 | 0.21 | 0.001 |
| DST-backwards **^a^** | 4.57 ± 1.29 | 4.00 ± 1.35 | 12.68 | <0.001 | 0.01 |
| **Episodic Memory (EM) ^a^** | -0.003 ± 2.25 | -3.05 ± 2.27 | 111.66 | <0.001 | 0.08 |
| AVLT-N1N3 **^a^** | 18.35 ± 4.87 | 12.20 ± 5.01 | 96.48 | <0.001 | 0.07 |
| AVLT-N4 **^a^** | 6.49 ± 2.35 | 3.37 ± 2.48 | 107.24 | <0.001 | 0.08 |
| AVLT-N5 **^a^** | 6.04 ± 2.49 | 2.84 ± 2.44 | 99.33 | <0.001 | 0.08 |
| **Processing Speed (PS) ^a^** | 0.01 ± 1.07 | -1.05 ± 1.88 | 45.16 | <0.001 | 0.04 |
| TMT A time (s) **^a^** | 58.83 ± 20.02 | 82.41 ± 40.73 | 65.05 | <0.001 | 0.05 |
| TMT B time (s) **^a^** | 164.40 ± 57.70 | 216.60 ± 101.24 | 36.20 | <0.001 | 0.03 |
| TMT B-A time (s) **^a^** | 106.19 ± 51.65 | 137.14 ± 74.50 | 14.73 | <0.001 | 0.01 |
| **Visuospatial Ability (VA) ^a^** | 0.07 ± 0.79 | -0.70 ± 1.31 | 56.11 | <0.001 | 0.04 |
| RO-delay **^a^** | 15.18 ± 6.99 | 10.65 ± 7.52 | 25.29 | <0.001 | 0.02 |
| RO-copy **^a^** | 33.98 ± 3.49 | 30.88 ±7.82 | 46.17 | <0.001 | 0.04 |
| **Language Ability (LA) ^a^** | -0.008 ± 1.30 | -0.92 ± 1.69 | 26.49 | <0.001 | 0.02 |
| CVFT-Animal **^a^** | 16.68 ± 6.49 | 14.02 ± 7.20 | 10.85 | 0.001 | 0.01 |
| CVFT-Vegetable **^a^** | 14.63 ± 5.75 | 13.01 ± 6.14 | 3.92 | 0.048 | 0.003 |
| CVFT-Fruit **^a^** | 11.45 ± 4.62 | 9.11 ± 4.55 | 13.27 | <0.001 | 0.01 |
| **General Cognitive Ability (GCA) ^a^** | 0.15 ± 4.23 | -6.05 ± 3.64 | 135.14 | <0.001 | 0.10 |

***Note.*** The above values are represented by the mean ± SD (IQR) or number (proportion %). ^a^ Adjusted for age. Abbreviations: GDS = Geriatric Depression Scale; LS = Loneliness Scale.

**eTable 3 β coefficients for the associations of LICR and Time with changes in cognitive function over the follow-up period.**

|  | **WM**  **Estimate (95% CI)** | **EM**  **Estimate (95% CI)** | **PS**  **Estimate (95% CI)** | **VA**  **Estimate (95% CI)** | **LA**  **Estimate (95% CI)** | **GCA**  **Estimate (95% CI)** |
| --- | --- | --- | --- | --- | --- | --- |
| **Longitudinal^a^** |  |  |  |  |  |  |
| Intercept | -0.008 (-0.06, 0.05) | 0.08 (-0.04, 0.21) | 0.02 (-0.04, 0.08) | -0.005 (-0.05, 0.04) | 0.02 (-0.05, 0.08) | 0.10 (-0.12, 0.33) |
| Time | 0.01 (-0.004, 0.03) | -0.11 (-0.15, -0.08)^***^ | -0.02 (-0.04, -0.002)^*^ | 0.001 (-0.01, 0.02) | -0.02 (-0.04, -0.005)^*^ | -0.15 (-0.21, -0.10)^***^ |
| **Longitudinal^b^** |  |  |  |  |  |  |
| Age | -0.01 (-0.02, -0.005)^**^ | -0.09 (-0.11, -0.07)^***^ | -0.05 (-0.06, -0.04)^***^ | -0.009 (-0.02, -0.002)^*^ | -0.02 (-0.03, -0.01)^***^ | -0.16 (-0.20, -0.13)^***^ |
| Time | 0.09 (-0.09, 0.26) | 0.41 (0.03, 0.80)^*^ | 0.25 (0.04, 0.45)^*^ | 0.16 (-0.001, 0.32)^+^ | 0.35 (0.14, 0.55)^**^ | 1.30 (0.72, 1.88)^***^ |
| Age × Time | -0.001 (-0.003, 0.002) | -0.006 (-0.01, -0.0002)^*^ | -0.003 (-0.006, 0.0003)^+^ | -0.002 (-0.004, 0.0002) | -0.005 (-0.008, -0.002)^**^ | -0.02 (-0.03, -0.009)^***^ |
| **Longitudinal^c^** |  |  |  |  |  |  |
| Time | 0.21 (-0.04, 0.46) | 0.32 (-0.24, 0.88) | 0.34 (0.05, 0.63)^*^ | 0.44 (0.21, 0.67)^***^ | 0.07 (-0.23, 0.36) | 1.45 (0.61, 2.29)^**^ |
| Continuous LICR | 0.005 (-0.02, 0.03) | 0.02 (-0.03, 0.07) | 0.03 (0.008, 0.06)^*^ | 0.02 (-0.003, 0.04) | -0.004 (-0.03, 0.02) | 0.04 (-0.04, 0.12) |
| Continuous LICR × Time | -0.004 (-0.02, 0.02) | -0.008 (-0.05, 0.03) | -0.01 (-0.03, 0.01) | 0.01 (-0.005, 0.03) | 0.03 (0.007, 0.05)^*^ | 0.02 (-0.04, 0.08) |

***Note.*** ^a^ was unconditional growth model, mainly focused on the changes of various cognitive functions over time. ^b^ added age to the unconditional growth model . ^c^ Adjusted for baseline age (and age × time), sex, hypertension (and hypertension × time), diabetes (and diabetes × time), hyperlipidaemia (and hyperlipidaemia × time), coronary heart disease, cerebral small-vessel disease, smoking (and smoking × time), drinking (and drinking × time), BMI (and BMI × time), depression (and depression × time), loneliness (and loneliness × time) and physical activity (and physical activity × time) and corresponding baseline cognitive functions. Abbreviations: LICR = lifetime intellectual cognitive reserve; EA = educational attainment; OC = occupational complexity; MLA = mental leisure activity; WM = working memory; EM = episodic memory; PS = processing speed; VA = visuospatial ability; LA = language ability; GCA = general cognitive ability. ^***^ *p*< 0.001, ^**^ *p*< 0.01, ^*^ *p*< 0.05, ^+^ *p*< 0.065.

**eTable 4 Sex differences in β coefficients for the associations of the components of LICR with baseline and change of cognitive functions over the follow-up period.**

|  | **WM**  **Estimate (95% CI)** | **EM**  **Estimate (95% CI)** | **PS**  **Estimate (95% CI)** | **VA**  **Estimate (95% CI)** | **LA**  **Estimate (95% CI)** | **GCA**  **Estimate (95% CI)** |
| --- | --- | --- | --- | --- | --- | --- |
| **Men** |  |  |  |  |  |  |
| **Baseline^a^** |  |  |  |  |  |  |
| Continuous EA | 0.11 (0.07, 0.15)^***^ | 0.10 (0.02, 0.17)^**^ | 0.11 (0.08, 0.15)^***^ | 0.04 (0.01, 0.08)^**^ | 0.04 (0.01, 0.08)^*^ | 0.41 (0.28, 0.53)^***^ |
| Continuous OC | 0.02 (-0.01, 0.06) | 0.07 (0.01, 0.13)^*^ | 0.07 (0.04, 0.10)^***^ | 0.01 (-0.02, 0.04) | 0.03 (-0.01, 0.06) | 0.20 (0.09, 0.31)^***^ |
| Continuous MLA | 0.01 (0.004, 0.02)^**^ | 0.01 (-0.01, 0.03) | 0.02 (0.01, 0.02)^***^ | 0.01 (0.001, 0.01)^*^ | 0.01 (0.004, 0.02)^**^ | 0.06 (0.03, 0.09)^***^ |
| **Longitudinal^b^** |  |  |  |  |  |  |
| Continuous EA × Time | -0.007 (-0.02, 0.01) | 0.005 (-0.03, 0.04) | 0.002 (-0.01, 0.02) | 0.01 (-0.001, 0.03) | 0.006 (-0.01, 0.02) | 0.02 (-0.02, 0.07) |
| Continuous OC × Time | -0.004 (-0.02, 0.01) | -0.005 (-0.03, 0.02) | -0.01 (-0.02, 0.002) | 0.006 (-0.006, 0.02) | 0.003 (-0.01, 0.02) | -0.01 (-0.05, 0.03) |
| Continuous MLA × Time | 0.0001 (-0.003, 0.003) | -0.0005 (-0.007, 0.01) | 0.002 (-0.002, 0.005) | 0.0001 (-0.003, 0.003) | -0.002 (-0.006, 0.002) | -0.0009 (-0.01, 0.01) |
| **Women** |  |  |  |  |  |  |
| **Baseline^a^** |  |  |  |  |  |  |
| Continuous EA | 0.10 (0.08, 0.13)^***^ | 0.15 (0.10, 0.21)^***^ | 0.11 (0.08, 0.13)^***^ | 0.06 (0.04, 0.08)^***^ | 0.06 (0.03, 0.08)^***^ | 0.48 (0.39, 0.57)^***^ |
| Continuous OC | 0.03 (0.01, 0.05)^***^ | 0.07 (0.03, 0.11)^***^ | 0.04 (0.02, 0.06)^***^ | 0.02 (0.002, 0.03)^*^ | 0.02 (-0.001, 0.04) | 0.17 (0.11, 0.24)^***^ |
| Continuous MLA | 0.01 (0.006, 0.02)^***^ | 0.03 (0.02, 0.04)^***^ | 0.01 (0.008, 0.02)^***^ | 0.01 (0.003, 0.01)^***^ | 0.01 (0.005, 0.02)^***^ | 0.07 (0.05, 0.09)^***^ |
| **Longitudinal^b^** |  |  |  |  |  |  |
| Continuous EA × Time | 0.0002 (-0.01, 0.01) | -0.003 (-0.03, 0.02) | -0.0004 (-0.01, 0.01) | 0.001 (-0.008, 0.01) | 0.019 (0.008, 0.03)^**^ | 0.02 (-0.02, 0.05) |
| Continuous OC × Time | 0.001 (-0.006, 0.01) | -0.003 (-0.02, 0.01) | -0.006 (-0.01, 0.002) | 0.005 (-0.001, 0.01) | 0.008 (-0.0003, 0.02)^+^ | 0.006 (-0.02, 0.03) |
| Continuous MLA × Time | -0.0008 (-0.003, 0.001) | -0.002 (-0.006, 0.002) | -0.0004 (-0.002, 0.002) | 0.0002 (-0.002, 0.002) | 0.0005 (-0.002, 0.003) | -0.002 (-0.008, 0.004) |

***Note.*** ^a^ Adjusted for baseline age (and age × time), sex, hypertension (and hypertension × time), diabetes (and diabetes × time), hyperlipidaemia (and hyperlipidaemia × time), coronary heart disease, cerebral small-vessel disease, smoking (and smoking × time), drinking (and drinking × time), BMI (and BMI × time), depression (and depression × time), loneliness (and loneliness × time) and physical activity (and physical activity × time). ^b^ Adjusted for the variables in ^a^ and corresponding baseline cognitive functions. Abbreviations: LICR = lifetime intellectual cognitive reserve; EA = educational attainment; OC = occupational complexity; MLA = mental leisure activity; WM = working memory; EM = episodic memory; PS = processing speed; VA = visuospatial ability; LA = language ability; GCA = general cognitive ability. ^***^ *p*< 0.001, ^**^ *p*< 0.01, ^*^ *p*< 0.05, ^+^ *p*< 0.065.

# Supplementary reference

1.	Du C, Li X, Li J, Wang W, Dang M, Cheng J, et al. Leisure activities as reserve mediators of the relationship between loneliness and cognition in aging. Transl psychiat. 2024;14(1):1-6. <https://doi.org/10.1038/s41398-024-02960-6>.

2.	Russell DW. UCLA Loneliness Scale (Version 3): Reliability, Validity, and Factor Structure. J Pers Assess. 1996;66(1):20-40. <https://doi.org/10.1207/s15327752jpa6601_2>.

3.	Dai X, Liu S, Li X, Chen K, Gao S, Wang J, et al. Longitudinal association between depressive symptoms and cognitive function: the neurological mechanism of psychological and physical disturbances on memory. Psychol med. 2024;54(13):3602-11. <https://doi.org/10.1017/S0033291724001612>.

4.	Yesavage JA, Blind T, Rose TL. Development and validation of a geriatric depression screening scale: a preliminary report. J Psych Res. 1982;17(1):37-49. <https://doi.org/10.1016/0022-3956(82)90033-4>.
